# Assessing Cross-Contamination in Spike-Sorted Electrophysiology Data

**DOI:** 10.1523/ENEURO.0554-23.2024

**Published:** 2024-08-20

**Authors:** Jack P. Vincent, Michael N. Economo

**Affiliations:** ^1^Department of Biomedical Engineering, Boston University, Boston, Massachusetts 02215; ^2^Center for Neurophotonics, Boston University, Boston, Massachusetts 02215; ^3^Center for Systems Neuroscience, Boston University, Boston, Massachusetts 02215

**Keywords:** electrophysiology, spike sorting

## Abstract

Recent advances in extracellular electrophysiology now facilitate the recording of spikes from hundreds or thousands of neurons simultaneously. This has necessitated both the development of new computational methods for spike sorting and better methods to determine spike-sorting accuracy. One long-standing method of assessing the false discovery rate (FDR) of spike sorting—the rate at which spikes are assigned to the wrong cluster—has been the rate of interspike interval (ISI) violations. Despite their near ubiquitous usage in spike sorting, our understanding of how exactly ISI violations relate to FDR, as well as best practices for using ISI violations as a quality metric, remains limited. Here, we describe an analytical solution that can be used to predict FDR from the ISI violation rate (ISI_v_). We test this model in silico through Monte Carlo simulation and apply it to publicly available spike-sorted electrophysiology datasets. We find that the relationship between ISI_v_ and FDR is highly nonlinear, with additional dependencies on firing frequency, the correlation in activity between neurons, and contaminant neuron count. Predicted median FDRs in public datasets recorded in mice were found to range from 3.1 to 50.0%. We found that stochasticity in the occurrence of ISI violations as well as uncertainty in cluster-specific parameters make it difficult to predict FDR for single clusters with high confidence but that FDR can be estimated accurately across a population of clusters. Our findings will help the growing community of researchers using extracellular electrophysiology assess spike-sorting accuracy in a principled manner.

## Significance Statement

High-density silicon probes are widely used to record the activity of large populations of neurons while animals are engaged in complex behavior. In this approach, each electrode records spikes from many neurons, and “spike-sorting” algorithms are used to group the spikes originating from each neuron together. This process is error-prone, however, and so the ability to assess spike-sorting accuracy is essential for properly interpreting neural activity. The rate of interspike interval (ISI) violations is commonly used to assess spike-sorting accuracy, but the relationship between the ISI violation rate (ISI_v_) and sorting accuracy is complex and poorly understood. Here, we describe this relationship in detail and provide guidelines for how to properly use ISI_v_ to assess spike-sorting accuracy.

## Introduction

Extracellular electrophysiology has become an increasingly popular method for studying neuronal activity at the population level. Silicon probes containing dozens or hundreds of densely packed electrode sites can be used to observe neuronal action potentials in many neurons simultaneously. This multiplexed signal acquisition, however, often necessitates the assignment of observed action potentials to individual neurons—“spike sorting”—as a critical first step prior to many analyses (Todorova et al., 2014; Rey et al., 2015). Approaches for spike sorting vary, though all generally involve comparisons of observed action potential waveforms within and across electrodes and then grouping similar spikes—thought to be produced by the same neuron—together, forming clusters (Gibson et al., 2012; Quiroga and Panzeri, 2013). Ideally, each cluster is composed principally of true-positive (TP) spikes from a single neuron making it a “well-isolated” cluster. Spikes from this single neuron can be erroneously excluded from the cluster, resulting in false negatives (FNs). The primary focus of this work, however, is false positives (FPs), wherein spikes are misassigned to a cluster whose activity is meant to correspond to a different neuron. Clusters with substantial “contamination” by FPs thus represent the combined activity of multiple neurons.

FPs are a persistent problem in spike sorting that results from frequently unavoidable similarities in action potential waveforms, occurrence of spikes at overlapping times, nonstationarity in waveform shape, and recording noise. Contamination can distort the activity of a cluster, potentially leading to incorrect conclusions about how single neurons encode information. For instance, a population of neurons recorded during a task with two cues may contain neurons responsive to just one cue or the other. However, poor sorting may lead to cluster cross-contamination between these two phenotypes, giving the impression that neurons in this region respond to both cues. The prevalence of FPs in a single cluster, recording session, or dataset can be described by the false discovery rate (FDR), a value that ranges between 0 and 1 and reports the proportion of sorted spikes that have been misassigned. While FNs can also be a concern, as they can decrease recorded spike frequencies and thus reduce statistical power in subsequent analyses (Hill et al., 2011), FNs should not generally alter the overall patterns of activity associated with individual clusters and are thus of less concern than FPs.

Algorithmic approaches to spike sorting for high-density silicon probe electrophysiology have seen concentrated development over the last decade, with researchers now selecting from a number of competing options for high-throughput automated spike sorting (Bestel et al., 2012; Pachitariu et al., 2016; Chung et al., 2017; J. H. Lee et al., 2017; Jun et al., 2017; Chaure et al., 2018; Buccino et al., 2020; Saif-ur-Rehman et al., 2021; Toosi et al., 2021), yet concomitant techniques for post hoc analysis of spike-sorted data (Pouzat et al., 2002; Hill et al., 2011; Neymotin et al., 2011; Barnett et al., 2016; Magland et al., 2020) have been given comparatively less attention. Sorting quality metrics generally fall into two categories: assessment of cluster overlap using dimensionally reduced representations of sorted spike waveforms, e.g., *L*-ratio (Schmitzer-Torbert et al., 2005), isolation distance (Harris et al., 2001), *D*-prime (Hill et al., 2011), and silhouette score, or empirical measures known to be related to cluster isolation and sorting difficulty, e.g., signal-to-noise ratio, presence ratio, firing range, and interspike interval (ISI) violations. Among all these metrics, ISI violations are unique in that they are tightly linked to the occurrence of FPs. Biophysical limitations prevent neurons from producing consecutive spikes within their absolute refractory period, meaning the presence of action potentials spaced by less than the absolute refractory period, an ISI violation, is always the result of at least one FP.

ISI violations are typically reported as a fraction of the total number of spikes assigned to a cluster. The ISI violation rate (ISI_v_)—the number of ISI violations divided by the total number of spikes assigned to a cluster—is often interpreted subjectively with only a general understanding that a lower ISI_v_ is associated with a lower FDR. Often, although not always, it is appreciated that most FP spikes do not produce ISI violations and so ISI_v_ << FDR. Previous work has estimated the relationship between ISI_v_ and FDR (Hill et al., 2011; Llobet et al., 2022) using simplifying assumptions, but the accuracy and limitations of predicting cluster FDR on the basis of ISI violations under realistic experimental conditions have not been assessed.

Here, we develop a comprehensive model explaining the relationship between ISI_v_ and FDR following spike sorting with respect to cluster contamination, neuronal firing frequency, the temporal relationships between neurons, and the number of neurons contributing FPs. We benchmark the accuracy of this model in silico through Monte Carlo simulation and explore limitations in the accuracy of FDR estimation imposed by in vivo recording conditions. Finally, we apply this model to publicly available spike-sorted electrophysiology data to estimate FDRs from ISI violations in published studies and provide researchers with intuition about the range of FDRs expected in silicon probe electrophysiology.

## Materials and Methods

### Monte Carlo simulation of neural spike trains

Stochastic neural spike trains were simulated using Elephant (Electrophysiology Analysis Toolkit; Yegenoglu et al., 2018). Specifically, neurons were modeled as either homogeneous or inhomogeneous Poisson processes (van Vreeswijk, 2010) using either the StationaryPoissonProcess or the NonStationaryPoissonProcess functions of the spike_train_generation module. Custom Python scripts were used for subsequent simulation and analysis. While Poisson point processes were used for all simulations of neural spike trains, post hoc custom simulations of gamma, inverse Gaussian, and log-normal point processes indicated that observed results did not seem to depend on any particular point process or ISI coefficient of variation (CV; Extended Data Fig. 3-1). A refractory period of 2.5 ms was assumed for all simulations as well as all calculations in Table 2. All datasets examined were recorded in mice of both sexes, and most recordings targeted the neocortex. However, for data collected from different brain regions or species, this parameter would need to be adjusted accordingly. Simulating “infinite” contaminant neurons was accomplished by simulating a single contaminant neuron with no refractory period. Simulated recording durations varied depending on the desired level of certainty in ISI_v_ and need to emulate realistic recording conditions. These times included ∼28 h (Fig. 3*A*,*B*), ∼17 h (Fig. 3*C*), 12 h (Fig. 3*D*), 30 min (Fig. 2*B*), and 10 min (Figs. 2*A*,*C*, 4*B*,*C*).

### Monte Carlo simulation of cluster populations

**Figure 1.**
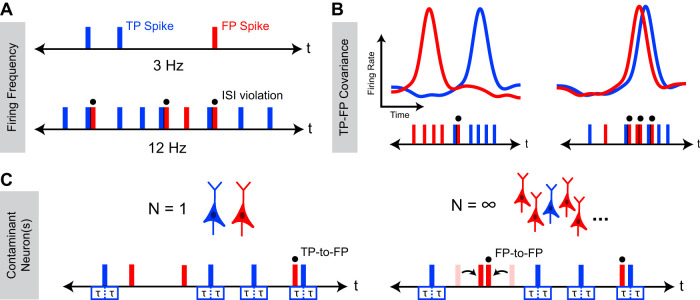
Factors affecting the relationship between ISI_v_ and FDR. Schematic representation of occurrence of ISI violations for a cluster with varying firing frequencies (***A***) TP–FP covariance (***B***) and numbers of contaminant neuron(s) (***C***). In all cases, underlying FDR between the two cases is the same, while observed ISI_v_ varies as a consequence of changes in these characteristics of neuronal activity. Blue corresponds to TP spikes, and red corresponds to FP spikes. Overhead dots indicate observed ISI violations. *τ* represents the neuronal refractory period. ISI violations can occur between TPs and FPs (TP-to-FP) or, if multiple contaminant neurons are present, FPs and other FPs (FP-to-FP).

In some simulations, all parameters were varied simultaneously to produce populations of clusters with a wide range of physiologically feasible parametric combinations (Figs. 3*D*, 4*B*,*C*). For each cluster, the following multistep process was used: (1) FDR, *N*, and *f*_t_ were randomly sampled from bounded probability distributions. Specifically, FDR was sampled from bounded Cauchy distributions as these were found to most accurately replicate ISI_v_ distributions found in electrophysiological data when simulated. Different population median and mean FDRs were obtained by adjusting the location and scale of the Cauchy distribution. *f*_t_ was randomly selected from a bounded uniform distribution: [4, 20] (Fig. 3*D*), [1, 10] (Fig. 4*B*), and [4, 16] (Fig. 4*C*). Possible values of *N* were uniformly chosen from among the following values: [1, 2, 5, ∞]. This was found to be the most balanced way to sample *N*, as the effect of contaminant neuron count on ISI violation occurrence increases logarithmically. (2) Unit vector peristimulus time histograms (PSTHs) were randomly selected from one of the datasets in Table 2 to serve as $\;{\hat{f}}_{\mathrm{TP}}$ and $\;{\hat{f}}_{\mathrm{FP}}$. These were selected uniformly from among all clusters present in the dataset. (3) These PSTHs were then scaled appropriately and combined to reach the desired *f*_t_ and FDR. The correct scaling was determined using a function optimization routine (minimize_scalar, SciPy) that sought to minimize the difference between each scaled PSTH's current and desired average firing frequency. TP–FP covariance obtained using this method varied from −49.2 to 113.7 Hz^2^. Longer simulated recording times were attained through repeated simulation of these PSTHs.

### Calculation of PSTHs

PSTHs of cluster responses for in vivo electrophysiology data were calculated using bin sizes of 50 ms. Predicted median and mean FDR were found to be largely unaffected by bin size selection in trial-averaged PSTHs. For continuously recorded data, the greatest length of time that could be extracted around each cue without overlapping between “trials” was used to generate trial-averaged PSTHs.

### Estimating unknown cluster parameters

When predicting FDR using recorded data, only limited information is available about each sorted cluster. ${\vec{\;f}}_{t}$ and ISI_v_ can be computed directly. *τ* can be obtained through prior knowledge of electrophysiological properties of neurons in the organism and brain region being recorded from. $\;{\hat{f}}_{\mathrm{FP}}$ can be estimated through examination of other sorted clusters present in the recording session. Estimating *N* is not straightforward.

**Figure 2.**
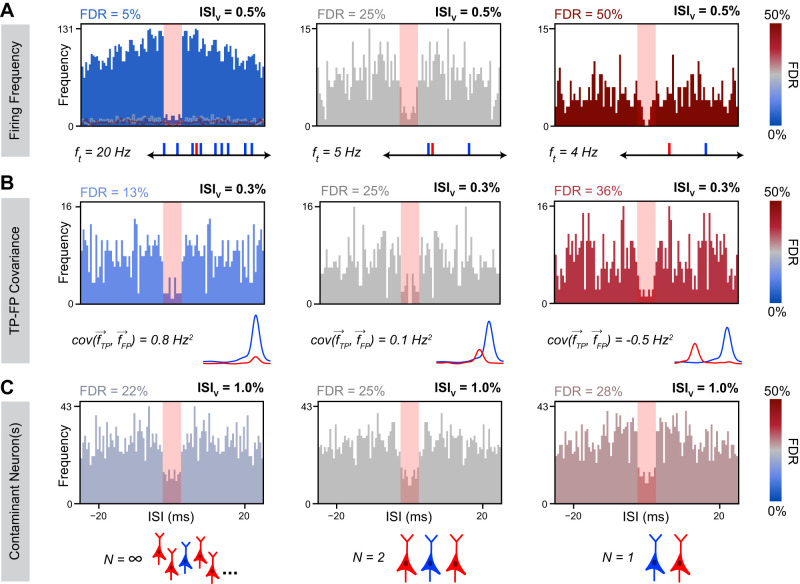
Simulated ISI distributions with variable underlying neuronal characteristics. Representative ISI histograms with all factors kept constant except total cluster firing frequency (***A***), TP–FP covariance (***B***) and contaminant neuron count (***C***). ISIs within the red rectangle are shorter than the refractory period and correspond to ISI violations. ISI_v_ was identical across conditions in each row. In ***A***, ISI violations may appear to constitute a smaller proportion of total ISIs in higher firing frequency conditions, but this is only because these conditions had lower average ISIs and all conditions were plotted within the same 0–25 ms ISI domain. Simulated firing frequencies were as indicated (***A***), 2 Hz (***B***), and 10 Hz (***C***). Simulated firing was either homogeneous (***A***, ***C***) or inhomogeneous (***B***). In ***A***, outlines of the histograms in the right two panels are overlaid to scale with high transparency on the left panel. Blue corresponds to TP spikes, and red corresponds to FP spikes. FP firing frequency traces (***B***) are schematics only. The case of “infinite” contaminant neurons was simulated using a single contaminant neuron with no refractory period.

To account for uncertainty in *N*, two approaches can be taken. A reasonable guess (e.g., 2, 3) can be chosen, and simply plugged directly into the equation, or multiple values representing extreme cases can be input and their results averaged. For example, FDR can be calculated by assuming *N* equals either 1 or ∞ and then taking the average of results from these two cases. Empirically, we found this equivalent to assuming a single *N* of ∼2–3. Our results indicate that FDR estimates are not highly sensitive to the particular choice of *N* (Fig. 2*C*).

Depending on the assumed *N*, $\;{\hat{f}}_{\mathrm{FP}}$ can be estimated from either other single clusters or averaged combinations of clusters. For example, if an *N* of 1 is assumed, $\;{\hat{f}}_{\mathrm{FP}}$ can be directly obtained from other random clusters, while if an *N* of 2 is assumed, $\;{\hat{f}}_{\mathrm{FP}}$ can be obtained by taking the average of 2 other random clusters. If an *N* of ∞ is assumed, $\;{\hat{f}}_{\mathrm{FP}}$ can be obtained from a global average PSTH. Clusters for deriving $\;{\hat{f}}_{\mathrm{FP}}$ can be restricted to those on the same or nearby electrode sites.

### Estimation of FDR in simulated cluster populations and electrophysiology datasets

**Figure 3.**
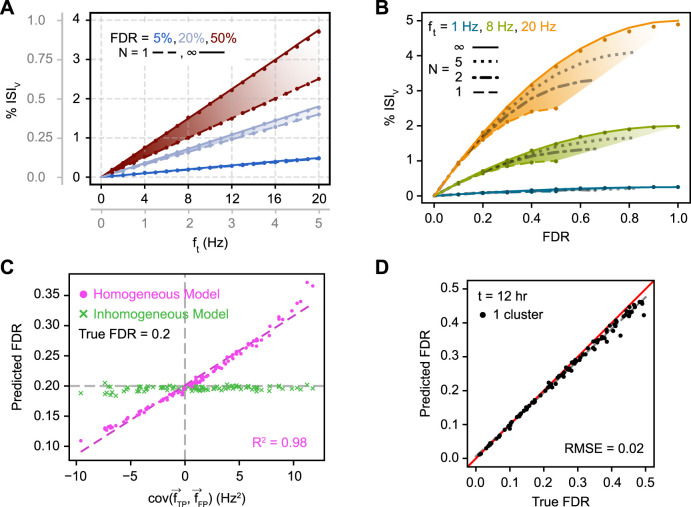
Relationship between single-cluster FDR and observed ISI_v_. ***A***, Dependence of ISI_v_ on total firing frequency given varying FDRs and contaminant neuron counts. Lines correspond to analytical predictions; dots correspond to simulation results. Plotted data apply to both primary and gray axes; the latter provide an inset for the former. ***B***, Dependence of ISI_v_ on FDR given varying firing frequencies and contaminant neuron counts. Conventions as in ***A***. ***C***, Prediction of FDR from observed ISI_v_ with temporally inhomogeneous firing frequencies using either the homogeneous model (Eq. 9) or the inhomogeneous model (Eq. 10). ***D***, Prediction of FDR from observed ISI_v_ for 100 total clusters simulated across a range of physiologically relevant underlying neuronal characteristics. Total firing frequency was varied between 4 and 20 Hz, *N* was varied between 1 and ∞, and $\;{\hat{f}}_{\mathrm{FP}}$ was obtained by averaging across other clusters (see Materials and Methods for more details). The red line is the unity line, or perfect concurrence between predicted and true FDR; dashed gray line is the line of best fit. RMSE calculated with respect to the unity line. Clusters shown in ***D*** simulated using Poisson point processes. See Extended Data Figure 3-1 for validation using non-Poisson point processes. Simulated firing was either homogeneous (***A***, ***B***) or inhomogeneous (***C***, ***D***). The model used for FDR predictions was either homogeneous (***A***, ***B***) or inhomogeneous (***D***), or both were used (***C***).

**Figure 4.**
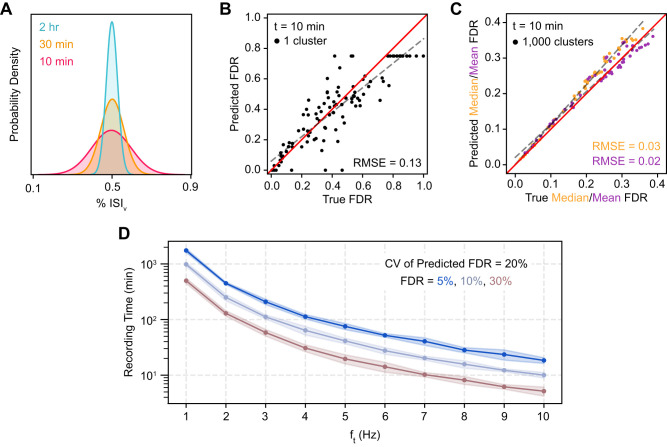
Prediction of FDR for single clusters and populations. ***A***, Probability density functions of observed % ISI_v_ for a prototypical 8 Hz, 15% FDR cluster recorded for varying time lengths. These parameters produce a true % ISI_v_ of 0.5%, a value that has been used as a threshold for considering clusters well isolated (Jadhav et al., 2009; Guo et al., 2014; Chandrasekaran et al., 2017). ***B***, Prediction of FDR in single clusters recorded for 10 min. All parameters varied simultaneously across a range of physiologically relevant underlying neuronal characteristics. Total firing frequency was varied between 1 and 10 Hz, *N* was varied between 1 and ∞, and $\;{\hat{f}}_{\mathrm{FP}}$ was obtained by averaging across other clusters (see Materials and Methods for more details). Ceiling effect of predicted FDR at 0.75 due to theoretical limit of FDR when predicting with unknown *N* (see Materials and Methods). The red line is the unity line or perfect concurrence between predicted and true FDR; the dashed gray line is the line of best fit. RMSE calculated with respect to the unity line. ***C***, Prediction of median and mean FDR in 1,000-cluster populations. Cluster FDRs are Cauchy-distributed around the population mean. Total firing frequency was varied between 4 and 16 Hz; otherwise parameters varied simultaneously and conventions as in ***B***. ***D***, Minimum recording time required for predictions of FDR in a single cluster to have a CV of 20%.

For FDR predictions in both simulated cluster populations (Figs. 3*D*, 4*B*,*C*) and electrophysiology datasets (Table 2), the following methodology was used. An *N* of both 1 and ∞ were assumed: in the former case, a given sorted cluster was compared with every other cluster in the simulation or recording session, while in the latter, it was compared with a single global $\;{\hat{f}}_{\mathrm{FP}}$. The final FDR is the mean of the (*N* = 1) cases averaged with the (*N* = ∞) case.

For estimating FDR in electrophysiology datasets (Table 2), clusters for deriving $\;{\hat{f}}_{\mathrm{FP}}$ were not restricted to those on the same or nearby electrode sites due to uncertainty about each dataset's probe geometry. Probe geometry was also not implemented in simulations of cluster populations (Figs. 3*D*, 4*B*,*C*). A single *τ* of 2.5 ms was used for every dataset, all of which were comprised of recordings from mouse brains. This refractory period was modified by the censor period, when necessary. Censor periods were determined through visual inspection of aggregated per-cluster ISI histograms across the entire dataset. For selecting datasets to examine, only papers published in the last 10 years with publicly available spike-sorted electrophysiology data were considered. No limits or minimums were placed on cluster count, and no sorting methods were specifically included or excluded. In some datasets, spontaneous activity was recorded without a cue to calculate PSTHs around. In such cases, FDR was initially predicted using firing frequency vectors calculated across the entire session. However, these predictions were found to be sensitive to the bin size used; therefore the predicted FDR assuming homogeneous firing is given instead (Table 1; Eq. 9).

**Table 1.** Symbols used

Symbol	Description	Units
FDR	False discovery rate	Unitless or %
ISI_v_	Interspike interval violation rate	Unitless or %
*f*_t_	Total observed firing frequency	Hz
*f*_TP_	Frequency of TP spikes	Hz
*f*_FP_	Frequency of FP spikes	Hz
*f*_v_	Frequency of ISI violations	Hz
*τ*	Neuronal refractory period	s
*τ*_c_	Spike-sorting censor period	s
*τ*_e_	Effective refractory period	s
*N*	Number of contaminant neurons	-
*n*	Number of elements in a firing frequency vector	-

### Theoretical limits of predicted FDR

Due to stochasticity associated with ISI_v_ estimates, observed ISI_v_ values sometimes exceed theoretical bounds given by the chosen parameters, producing imaginary predicted FDRs. In such cases, the predicted FDR is capped at its theoretical maximum for a given (assumed) number of contaminant neurons (Eq. 1). This maximum is derived from the fact that given a certain *N*, if predicted FDR exceeds FDR_max_, a lower FDR could be attained by simply selecting a different neuron within the sorted cluster to be the TP neuron. When FDR is being calculated by averaging the (*N* = 1) and (*N* = ∞) cases, FDR_max_ is the average of these two cases’ maximums, 0.75, as follows:

$$\begin{array}{c} \mathrm{FD}R_{\max}=\frac{N}{N+1} \end{array}.$$

### Code availability

The code for calculating FDR with user-provided spike–sorted electrophysiology data, reproducing figures, and simulating neuronal firing and spike sorting is available via GitHub (https://github.com/economolab/DCISIv).

## Results

### The relationship between ISI_v_ and FDR is complex

We sought to understand how the occurrence of ISI violations depends on underlying cluster FDR and how other underlying characteristics of neuronal activity might affect this relationship. To this end, we focused on three variables likely to have a meaningful effect on the occurrence of ISI violations: neuronal firing frequency (Fig. 1*A*), temporal correlation of activity among the recorded population of neurons (Fig. 1*B*), and the number of contaminant neurons (Fig. 1*C*).

To determine how these variables affect the relationship between ISI_v_ and FDR, we used Monte Carlo simulations of neural spiking to examine how the relationship between ISI_v_ and FDR might change as a consequence of varying each parameter in isolation. Total cluster firing frequency was found to have a dramatic effect on ISI violation production, since overall firing frequency played a large role in determining the likelihood that any given FP spike would produce an ISI violation. Critically, clusters with lower firing frequencies and clusters with higher firing frequencies could both present with the same ISI_v_, even when they had markedly different underlying FDRs (5–50%; Fig. 2*A*). These results indicated a nonlinear relationship between ISI violation production and firing frequency not accounted for by simply dividing the number of ISI violations by the number of spikes (ISI_v_). The temporal overlap between TPs and FPs was also found to strongly modulate ISI violation production, although not as strongly as cluster firing frequency (Fig. 2*B*). Variable TP–FP covariance (0.8 to −0.5 Hz^2^) altered the probability of any given FP leading to an ISI violation, resulting in three clusters with substantially different FDRs (13–36%) presenting with the same ISI_v_ and firing frequency. Lastly, the number of contaminant neurons was also found to modulate ISI violation production, although not as meaningfully as other variables (Fig. 2*C*). Greater numbers of contaminant neurons increased the odds of ISI violations between pairs of FPs, meaning the same observed ISI_v_ was associated with a slightly lower FDR in cases of multiple contaminant neurons versus just one contaminant neuron. The dependence of this phenomenon on FP-to-FP violations (Fig. 1*C*) means its effects only became meaningful at high FDRs (>0.25) and total firing frequencies (>10 Hz).

### Analytical model of the relationship between ISI_v_ and FDR

We next sought to derive an analytical model describing the dependence of underlying FDR on observed ISI_v_ that incorporates each of these variables. To that end, we first considered a simplified case in which two neurons are each firing homogeneously or at a constant frequency and spikes from both neurons are being assigned to the same cluster. Each TP spike produces a double-sided “violation window” in time. If an FP spike occurs within that window, an ISI violation is observed (Eq. 2; Fig. 1*C*) as follows:

$$\begin{array}{c} \;f_{v}=f_{\mathrm{FP}}(2\tau f_{\mathrm{TP}}) \end{array}.$$

Here, we represent the number of ISI violations observed per second as *f*_v_, the neuronal absolute refractory period as *τ*, and the frequencies with which the two neurons produce TP and FP spikes as *f*_TP_ (TP frequency) and *f*_FP_ (FP frequency). Note that *f*_TP_ and *f*_FP_ may not necessarily represent the total firing frequencies of the neurons producing the TP and FP spikes, only the frequencies at which spikes from those neurons are assigned to a sorted cluster. These can be one and the same, e.g., for a neuron contributing TPs with no FNs. Also note that *f*_TP_ and *f*_FP_ cannot be measured experimentally, instead representing unseen parameters of spike train generation. By making substitutions based on a few simple relationships (Eqs. 3–5), Equation 2 can be solved using the quadratic formula, defining FDR as a function of the following experimentally observable variables: ISI_v_, *τ*, and *f*_t_—the total observed firing frequency of the cluster (Eq. 6; see Extended Data for intermediate steps) as follows:

$$\begin{array}{c} \mathrm{IS}I_{v}=\frac{\;f_{v}}{\;f_{t}} \end{array},$$

$$\begin{array}{c} \mathrm{FDR}=\frac{\;f_{\mathrm{FP}}}{\;f_{t}} \end{array},$$

$$\begin{array}{c} \;f_{t}=f_{\mathrm{TP}}+f_{\mathrm{FP}} \end{array},$$

$$\begin{array}{c} \mathrm{FDR}=\frac{1}{2}\left(1-\sqrt{1-\frac{2\mathrm{IS}I_{v}}{\tau f_{t}}}\right) \end{array}.$$

The larger root is ignored, i.e., the term under the square root is subtracted and not added, because the neuron with the most spikes in the cluster is de facto considered the TP-contributing neuron. Some spike-sorting algorithms make use of a censor period, whereby spikes detected within a certain minimum distance, *τ*_c_, of another spike are ignored. In such cases, the size of the violation window produced by each TP spike is shortened by this censor period producing a new effective refractory period: *τ*_e _= *τ* − *τ*_c_. Implementation of *τ*_e_ produces Equation 7, which is equivalent to a rearranged form of the equation derived in Hill et al. (2011) as follows:

$$\begin{array}{c} \;f_{v}=f_{\mathrm{FP}}(2\tau_{e}f_{\mathrm{TP}}) \end{array}.$$

At the other extreme, consider a situation where the spikes comprising a cluster are generated by an infinite number of neurons. In reality, there can never be an infinite number of contaminant neurons, but this term is a convenient shorthand for describing the limiting case where there as many contaminant neurons as FPs. In this limiting case, any FP can produce an ISI violation with any other FP, necessitating the addition of a second term wherein FP spikes now produce double-sided violation windows as well. This term is scaled by a factor of one-half to prevent double counting of FPs producing ISI violations with one another. Implementation of this term produces Equation 8, which is equivalent to a rearranged form of the equation derived in Llobet et al. (2022). This equation can be solved for FDR using substitutions and the quadratic formula as previously as the following:

$$\begin{array}{c} \;f_{v}=f_{\mathrm{FP}}(2\tau_{e}f_{\mathrm{TP}})+\frac{1}{2}f_{\mathrm{FP}}(2\tau_{e}f_{\mathrm{FP}}) \end{array}.$$

For an unspecified number of contaminant neurons *N*, an additional scaling factor of (*N* − 1) / *N* can be added to the FP–FP ISI violations term (Eq. 9). This factor can be interpreted as the fraction of all FPs available for any given contaminant neuron's spikes to produce ISI violations with, e.g., 1/2 for *N* = 2 and 2/3 for *N* = 3. This equation, like previous iterations, can be rearranged to solve for FDR with an additional dependence added on *N* as follows:

$$\begin{array}{c} \;f_{v}=f_{\mathrm{FP}}(2\tau_{e}f_{\mathrm{TP}})+\frac{1}{2}\left(\frac{N-1}{N}\right)f_{\mathrm{FP}}(2\tau_{e}f_{\mathrm{FP}}) \end{array}.$$

In the more biologically relevant case of inhomogeneous firing, or neural spiking frequencies that vary over time, *f*_TP_, *f*_FP_, *f*_t_, and *f*_v_ can all be considered not as constants but as functions of time (i.e., vector quantities). While this spiking nonstationarity must be taken into account, a time-varying estimate of FDR would be a needless level of granularity and also highly inaccurate given the stochastic nature of neuronal spiking and ISI violations, so of primary interest is a time-averaged estimate of the relationship between violation frequency $\left({\bar{\;f}}_{v}\right)$ and underlying variables. In this case, the frequency of violations depends not on the product of the average values of *f*_TP_ and *f*_FP_ but on the expected value of their element-wise product, $E[{\vec{\;f}}_{\mathrm{TP}}{\vec{\;f}}_{\mathrm{FP}}]$, as follows:

$$\begin{array}{c} {\bar{\;f}}_{v}=2\tau_{e}E[{\vec{\;f}}_{\mathrm{TP}}{\vec{\;f}}_{\mathrm{FP}}]+\frac{1}{2}\left(\frac{N-1}{N}\right)2\tau_{e}E[{\vec{\;f}}_{\mathrm{FP}}{\vec{\;f}}_{\mathrm{FP}}] \end{array}.$$

For two vectors representing firing frequency over time ${\vec{\;f}}_{\mathrm{TP}}$ and ${\vec{\;f}}_{\mathrm{FP}}$ of length *n* elements, this expected value can be calculated as follows:

$$\begin{array}{c} E[{\vec{\;f}}_{\mathrm{TP}}{\vec{\;f}}_{\mathrm{FP}}]=\frac{{\vec{\;f}}_{\mathrm{TP}}\cdot {\vec{\;f}}_{\mathrm{FP}}}{n} \end{array}.$$

Equation 10 can then be solved for $\left|{\vec{\;f}}_{\mathrm{FP}}\right|$, the vector magnitude of ${\vec{\;f}}_{\mathrm{FP}}$, using the quadratic formula (Eq. 12). Unit vector $\;{\hat{f}}_{\mathrm{FP}}$ can subsequently be scaled by $\left|{\vec{\;f}}_{\mathrm{FP}}\right|$, averaged, and finally divided by the mean total firing frequency of the cluster to obtain a time-averaged estimate of FDR (Eq. 13) as follows:

$$\begin{array}{c} |{\vec{\;f}}_{\mathrm{FP}}|=\frac{N}{N+1}\left(D|{\vec{\;f}}_{t}|-\sqrt{D^{2}{\left|{\vec{\;f}}_{t}\right|}^{2}-\left(\frac{N+1}{N}\right)\left(\frac{{\bar{\;f}}_{t}\mathrm{IS}I_{v}n}{\tau_{e}}\right)}\right) \end{array},$$

$$\begin{array}{c} \mathrm{FDR}=\left(\frac{1}{{\bar{\;f}}_{t}}\right)\frac{1}{n}\sum \left(|{\vec{\;f}}_{\mathrm{FP}}|{\;\hat{f}}_{\mathrm{FP}}\right)=\frac{{\bar{\;f}}_{\mathrm{FP}}}{{\bar{\;f}}_{t}} \end{array}.$$

Here, *D* corresponds to the dot product of the unit vectors representing total cluster spike frequency and cluster FP spike frequency, $D=\;{\hat{f}}_{t}\cdot \;{\hat{f}}_{\mathrm{FP}}$. This can be thought of more generally as representing the degree to which the time-varying total cluster spike frequency temporally overlaps with the time-varying FP spike frequency. This final equation depends on a number of parameters specific to each cluster to obtain a single FDR estimate: (1) the effective refractory period *τ*_e_, (2) the temporal distribution of activity in the cluster of interest $\left({\vec{\;f}}_{t}\right)$ and (3) of other clusters contributing FP spikes $\left({\;\hat{f}}_{\mathrm{FP}}\right)$, (4) the observed ISI_v_, and (5) the number of contaminant neurons *N*. For information on how one can estimate these parameters from experimental data, see Materials and Methods.

### Prediction of FDR in silico

To both assess our model's predictive power and more generally illustrate relationships between model variables, we next simulated neural spike trains while varying all relevant parameters across a range of biologically relevant values and then attempted to predict FDR from the observed ISI_v_. In this case, parameters like *N* and $\;\;{\hat{f}}_{\mathrm{FP}}$ that may normally have associated uncertainty are known exactly. Simulating long periods of time (up to 28 h of recording time) also enables highly accurate estimates of ISI_v_.

For both homogeneous and inhomogeneous firing, we found that analytical FDR predictions closely approximated the true underlying FDRs (Fig. 3). For homogeneous firing specifically, observed ISI_v_ was found to strongly depend on FDR and total cluster firing frequency, as expected (Fig. 3*A*). A linear dependence was observed of ISI_v_ on firing frequency at fixed FDR and contaminant neuron counts, despite ISI_v_ often being assumed to already have normalized for cluster firing frequency. Furthermore, FDR was found to scale quadratically with increasing ISI_v_ at a fixed firing frequency and contaminant neuron count (Fig. 3*B*). ISI_v_ values of 0.1–1% represent typical thresholds in literature for considering a cluster well isolated (Jadhav et al., 2009; Roy and Wang, 2012; Chandrasekaran et al., 2017; Wright et al., 2021; Boucher et al., 2023; Zhao et al., 2023). Yet, our results indicate that FDRs associated with these ISI_v_ values vary considerably with the firing frequency of the cluster in question (Fig. 3*A,B*). As an illustration, an ISI_v_ of 0.5% reflected a desirable 5% FDR for a cluster firing at 20 Hz or a much higher 50% FDR for one firing at 3 Hz. In general, contaminant neuron count was of limited consequence unless the cluster in question had both a high firing frequency and high FDR, making FP–FP violations frequent enough to meaningfully affect overall ISI violation incidence.

10.1523/ENEURO.0554-23.2024.f3-1 Figure 3-1 **Prediction of FDR in non-Poisson point processes.** Prediction of FDR from observed ISI_v_ when simulating neural spiking by drawing ISIs from gamma distributions (**A**), inverse Gaussian distributions (**B**), or log-normal distributions (**C**). 100 total clusters simulated in each panel across a range of physiologically relevant underlying neuronal characteristics. Total firing frequency was varied between 4 and 20 Hz, N was varied between 1 and 10, and ${\hat{f}}_{FP}$ was obtained by averaging across other clusters (see *Materials and Methods* for more details). Coefficient of variation (CV) of ISI distributions varied from 0.5 to 2. Red line is the unity line, or perfect concurrence between predicted and true FDR; dashed gray line is the line of best fit. Root mean square error (RMSE) calculated with respect to the unity line. Simulated firing was homogeneous and the model used for FDR predictions was also homogeneous (*Eq. 9*). A gamma distribution with CV = 1 is identical to an exponential distribution, producing a Poisson point process. Bottom raster plots show gamma distributed spiking of an example 10 Hz neuron at various CVs. Stacked points indicate spikes occurring in quick succession. Download Figure 3-1, TIF file.

When the activity of clusters was inhomogeneous in time, errors in FDR predicted with the homogeneous model (Eq. 9) scaled linearly with the temporal covariance of TPs and FPs (Fig. 3*C*). Positive covariance increased the ISI_v_ at the same FDR, resulting in overestimation of FDR, while negative covariance conversely decreased ISI violations, resulting in underestimation of FDR. When inhomogeneous firing was appropriately taken into account (Eq. 10), predicted FDR closely approximated true FDR regardless of the covariances in neuronal firing. When the activity of clusters contributing TP spikes and FP spikes varied independently in time (inhomogeneous but zero covariance), the probability of TP–FP violations was unchanged compared with homogeneous spiking. Therefore, under these conditions, the homogeneous and inhomogeneous predictions agree even if the generation of TPs and FPs individually may not necessarily be homogeneous.

We next attempted to predict FDR from ISI_v_ (Eqs. 12, 13) in a population of clusters simulated inhomogeneously across a broad, randomized parameter space. Regardless of the precise combination of parameters for any given cluster, predicted FDR and true FDR agreed with low root mean square error (RMSE; Fig. 3*D*; RMSE, 0.02). This prediction accuracy also generalized to simulations of non-Poisson point processes across a range of possible ISI coefficients of variation (Extended Data Fig. 3-1; RMSE, 0.01–0.02).

### Prediction of FDR under realistic conditions

Our model performed well when ISI_v_ and other parameters were known exactly, although benchmarking simulations indicated that predictions of FDR are sensitive to small changes in ISI_v_ (Fig. 3), particularly at lower firing frequencies (Fig. 3*B*). We next wanted to determine whether FDR could be accurately predicted from recordings of finite duration with noisy ISI_v_ estimates and when exact values of *N* and $\;{\hat{f}}_{\mathrm{FP}}$ are unknown. Simulating spiking for finite durations, we found that observed ISI_v_ values were normally distributed around their true values (Fig. 4*A*), with increasing recording time decreasing the variance of this distribution, as expected.

To assess the effect of noisy ISI_v_ estimates on FDR predictions, we again simulated spiking while simultaneously varying all previously described parameters, but this time recording duration was restricted to 10 min (Fig. 4*B*,*C*). In this case, we found that FDR predictions for individual clusters were substantially less accurate (RMSE, 0.13), although they did not deviate systematically from true FDRs (Fig. 4*B*). Even with this highly restricted recording time, FDR population statistics could be estimated across a set of clusters with high fidelity so long as a sufficient number of clusters were sampled (Fig. 4*C*). For example, median FDR and mean FDR could be predicted with RMSEs of 0.03 and 0.02, respectively, across 1,000 clusters. Predicted median FDRs were slightly overestimated at high true median FDR (>0.25).

We next sought to determine the duration of neural recordings necessary to obtain accurate estimates of ISI_v_ and, thus, FDR in single clusters. To accomplish this, we simulated clusters with various firing frequencies and FDRs and determined the recording time sufficient to produce a CV of the predicted FDR of 20% (Fig. 4*D*; e.g., 50 ± 10% or 5 ± 1%). Surprisingly, we found that clusters with firing frequencies of 1–2 Hz required observations across hundreds of minutes to produce accurate estimates of single-cluster FDR, an infeasible recording time in many common experimental paradigms. For clusters with firing frequencies >5 Hz, FDRs between 5 and 30% could be estimated accurately using tens of minutes of spiking data.

We used our model to estimate the FDRs of clusters contained within 12 publicly available datasets that included spike-sorted electrophysiology recordings (Table 2). For all datasets, the inhomogeneous model (Eq. 10) was used, with the exception of Stringer et al. (2019) and Juavinett et al. (2019). Animal behavior in these datasets was not trial-based, and so it was not straightforward to accurately estimate time-varying firing frequencies, necessitating the use of the homogeneous model (Eq. 9; see Materials and Methods). Given limitations associated with single-cluster FDR predictions (Fig. 4*B*), estimated median and mean FDRs were reported across all clusters present in each dataset. An average median FDR of 12.9 ± 13.5% (SD) was observed along with an average mean FDR of 24.1 ± 9.2% (SD). No obvious correlations between cluster count, spike-sorting methodology, or recording technology and dataset FDR were observed. With the exception of Juavinett et al. (2019), median estimated FDRs were consistently lower than mean estimated FDRs. This implies that cluster FDR distributions in recorded electrophysiology datasets tend to be right-skewed, composed of a large proportion of clusters possessing FDRs closer to 0 as well as a broadly distributed complement of more contaminated clusters, some of which potentially reaching FDRs well above 0.5.

**Table 2.** Median and mean FDR of publicly available spike-sorted electrophysiology datasets

Authors	Median FDR (±SE)	Mean FDR (±SE)	# Clusters	τ*_c_* (ms)	Probe	Sorter
Xu et al. (2022)	3.1% (0.5)	14.3% (0.4)	3,046	0	H2/H3 *Cam. Neuro.*	Kilosort
Economo et al. (2018)	3.1% (0.6)	12.5% (0.5)	1,988	0.25	H2 *Cam. Neuro.*	JRClust
Steinmetz et al. (2019) ^a^	4.0% (0.2)	25.1% (0.2)	33,997	0	Neuropixels 1.0	Kilosort
Gao et al. (2018)	4.4% (0.7)	18.7% (0.6)	1,923	0.5	H2/A4 *Cam. Neuro./NeuroNexus*	UltraMegaSort2000
Li et al. (2016)	4.5% (0.8)	19.7% (0.7)	1,543	0.85	A4 *NeuroNexus*	UltraMegaSort2000
Inagaki et al. (2022)	6.2% (0.3)	17.7% (0.3)	7,968	0.25	HH-2/Neuropixels 2.0 *Janelia/N.A.*	JRClust/Kilosort
Guo et al. (2017)	6.5% (0.7)	18.8% (0.6)	1,936	0.85	A4/A2 *NeuroNexus*	UltraMegaSort2000
Finkelstein et al. (2021) ^a^	10.5% (0.5)	20.1% (0.4)	3,385	0.25	H2 *Cam. Neuro.*	JRClust
Sylwestrak et al. (2022)	12.8% (0.4)	29.1% (0.3)	10,548	0.25	Neuropixels 2.0	Kilosort
Stringer et al. (2019) ^b^	21.9% (0.5)	33.1% (0.4)	6,446	0	Neuropixels 1.0	Kilosort
Chinta and Pluta (2023)	28.2% (1.3)	36.2% (1.0)	991	0	*NeuroNexus*	Kilosort
Juavinett et al. (2019) ^a,b^	50.0% (0.8)	44.3% (0.7)	2,018	0	Neuropixels 1.0	Kilosort

a Clusters labeled “multi” or “MUA” excluded.

b Calculated using homogeneous model (Eq. 9).

Importantly, no attempts were made to curate clusters included in analysis from each dataset; all available sessions and clusters were assessed, with the exception of clusters labeled “multi” or “MUA” in datasets with cluster quality annotations. It's possible that some datasets were precurated, with clusters discarded according to some exclusion criteria prior to being uploaded, while others were shared uncurated. Depending on the nature of the scientific question being addressed, better cluster isolation is undoubtedly a larger priority for some datasets than others. For these reasons and due to uncertainty in assessing censor period in each dataset, we emphasize that these findings should serve generally as an overall survey of the range of expected contamination levels in datasets produced using widely used methods rather than as a commentary on individual datasets or spike-sorting methodologies.

## Discussion

ISI violations are the most commonly employed metric of accuracy in spike sorting, serving as an indication of the FDR—the rate at which spikes are erroneously assigned to the wrong cluster. Here, we used Monte Carlo simulations to demonstrate that the ISI_v_ is related to FDR through a complex relationship that depends on many factors, including (1) the neuronal firing frequency, (2) the temporal correlation in activity between neurons contributing to a cluster, and (3) the number of neurons contributing spikes to a cluster—in that order of descending importance. We derived an analytical model that can be used to predict FDR from ISI_v_ that incorporates these factors and determine the accuracy with which FDR can be inferred during finite-length recordings at the level of single clusters and datasets. Finally, we used this model to assess the FDR of clusters contained in publicly available spike-sorted electrophysiology datasets to provide bounds on the accuracy that can be reasonably expected by experimenters. Our study makes four central contributions.

First, we derive an analytical model that can be used to estimate FDR from ISI_v_ accurately across a broad parameter space.

Second, we explicitly demonstrate that FDR is not linearly related to ISI_v_ but depends critically on the total cluster spike frequency. While this dependence can be inferred from previous work (Hill et al., 2011; Llobet et al., 2022), our results underscore the inappropriateness of using ISI_v_ as an inclusion criterion for single clusters—which is still a common practice in many studies using spike-sorted data. Across a common range of firing frequencies (∼2 to 12 Hz), clusters with the same ISI_v_ can be associated with both low (∼5%) and very high (∼50%) FDRs (Figs. 2*A*, 3*A*).

Third, we find that estimates of FDR at the single-cluster level are noisy due to the stochasticity of ISI violations as well as uncertainty in cluster-specific parameters (Fig. 4*B*)—but estimates at the population level are highly robust (Fig. 4*C*). As a point of reference, our results suggest that ISI_v_ can be estimated accurately enough to predict single-cluster FDRs within 20% of their true values in 1 h recordings only when firing frequencies are greater than ∼5 Hz. Even for clusters that meet these requirements, however, experimental uncertainty in parameters like FP–TP covariance and contaminant neuron counts make single-cluster FDRs difficult to predict with high confidence. Alternatively, population-level statistics of FDR, obtained by averaging across all the clusters in a dataset, can be accurately predicted with recording durations as low as 10 min.

Finally, we predict FDR on the basis of ISI violations in publicly available datasets for the first time. FDR population statistics covered a wide range (median, 3.1–50.0%; mean, 12.5–44.3%) and could be estimated with low standard error (SE median, 0.2–1.3%; SE mean, 0.2–1.0%). Datasets with low FDR were not associated with any obvious external features of data collection, spike-sorting methodology, or recording technology, and the variance in FDRs across datasets is likely a function of the variable importance of low FDR for the scientific goals of individual studies.

### Monte Carlo simulations for validating prediction of FDR

Given the absence of high cluster count spike-sorted extracellular neural recordings with associated per-neuron ground truth patch–clamp data, Monte Carlo simulation presents itself as an attractive tool for studying the theoretical mechanisms by which a given level of contamination in a sorted cluster translates into observed ISI violations. Neural spiking was simulated in this work as a collection of independent Poisson processes, a common assumption that has been validated across a number of organisms, brain regions, and behavioral contexts (Werner and Mountcastle, 1965; Tolhurst et al., 1981; Shinomoto and Tsubo, 2001; Abbott and Dayan, 2005; Roxin et al., 2008). Furthermore, the equations derived in this work were equally accurate when applied to simulations of non-Poisson point processes across a range of ISI coefficients of variation, implying that they likely generalize to any renewal process that produces independent and identically distributed ISIs with a physiological CV (Extended Data Fig. 3-1). Beyond this foundation, only aspects of neuronal firing and spike sorting thought to potentially be relevant to ISI violation production were modeled, namely, varying firing frequency amplitudes, temporally inhomogeneous firing, and differing contaminant neuron counts. If an additional aspect not accounted for in this description plays a significant role in determining the relationship between ISI_v_ and FDR, then in silico validation may not reflect true congruence between analytical prediction and reality.

### Best practices for using ISI violations in spike sorting

When attempting to determine the success of spike-sorting operations post hoc, ISI violation fraction is frequently used as a per-cluster inclusion criterion. However, unless a cluster is recorded for a long-enough time period given its firing frequency and true FDR and difficult-to-estimate cluster–specific parameters are known, it can be difficult to predict FDR using ISI violations at the single-cluster level with high confidence. The use of ISI violation fractions in this way can easily result in situations where highly contaminated clusters are erroneously kept, while less contaminated clusters are discarded. We posit that the most straightforward and robust use case for ISI_v_ is as a tool for predicting population-level statistics of FDR when coupled with a sound theoretical understanding of how cluster contamination translates into ISI violations (Eqs. 10, 12, 13). Investigators can obtain an accurate estimate of median and mean cluster FDR across a session or dataset and then decide whether they are satisfied with these levels of cross-contamination or if additional effort to improve cluster isolation is needed.

When curating spike-sorted data, it is critical that both algorithmic and manual sorters do not specifically remove individual spikes that generate ISI violations. Typically, only a small fraction of contaminant spikes produce ISI violations; targeted removal of spikes producing ISI violations can reduce ISI_v_ substantially without meaningfully reducing the FDR, thus producing clusters that seem well isolated based on their ISI_v_, even when they are not. In effect, this practice does not accomplish anything except eliminating the predictive power of ISI_v_ for underlying FDR.

### Current state of spike sorting predicted using ISI violations

This work estimates an average median FDR of ∼13% and an average mean FDR of ∼24% in publicly available electrophysiology datasets. The lower median FDR compared with mean FDR across virtually all datasets examined indicates right-skewed FDR distributions. This likely arises as a consequence of FDR having a theoretical floor of 0, with most datasets having many cluster FDRs close to this floor. It may also be a consequence of most spike-sorting datasets being composed of two distinct types of clusters: relatively easier-to-sort clusters whose FDRs typically fall close to 0 and relatively harder-to-sort clusters whose FDRs are likely to fall more broadly over the theoretical range of FDR (0–1).

In the absence of any clear rationale for variance in FDR as predicted using ISI_v_ among publicly available spike-sorted datasets (Table 2), degree and execution of manual curation present themselves as promising explanatory candidates. While modern spike-sorting algorithms serve as an excellent basis for sorting vast quantities of electrophysiology data, many investigators still manually merge, split, and discard algorithmically obtained output clusters to further improve cluster isolation. Time, effort, and skill applied to manual curation are difficult to quantify and therefore unlikely to be reported in literature, although such differences are likely to have a material effect on the final quality of cluster isolation. The median and mean FDRs of the datasets examined here as well as the tendency toward right-skewed FDR distributions support the idea that all datasets are composed of both well and poorly isolated clusters.

### Necessity of high-quality spike sorting

It has been posited that well-sorted clusters are not a necessity for many types of neural data analyses, particularly those concerned with studying population dynamics (Christie et al., 2014; Trautmann et al., 2019). In some applications, however, well-isolated clusters remain a critical precondition for answering relevant neuroscientific questions. Characterizing the responsivity of specific cell types that have been identified on the basis of genetic expression, projection target, waveform shape, or activity in vivo represents an expansive line of inquiry wherein high-quality cluster isolation is key (Estebanez et al., 2017; Deubner et al., 2019; E. K. Lee et al., 2021; Ding et al., 2022; Takatoh et al., 2022). Ultimately, the level of cluster isolation necessary for a given study is highly dependent upon the biological questions of interest. The work herein aims to clarify the relationship between ISI violations and cluster contamination, as well as provide a tool by which overall spike-sorting quality can be quickly assessed with a direct, interpretable, and accurate metric, thereby streamlining assessments of sorting performance and increasing confidence that desired cluster isolation levels have been reached.

## Synthesis

Reviewing Editor: Nicholas J. Priebe, The University of Texas at Austin Decisions are customarily a result of the Reviewing Editor and the peer reviewers coming together and discussing their recommendations until a consensus is reached. When revisions are invited, a fact-based synthesis statement explaining their decision and outlining what is needed to prepare a revision will be listed below. The following reviewer(s) agreed to reveal their identity: Lauri Nurminen, Ivo Vanzetta. Note: If this manuscript was transferred from JNeurosci and a decision was made to accept the manuscript without peer review, a brief statement to this effect will instead be what is listed below.

SYNTHESIS

Two reviewers evaluated the manuscript "Assessing cross-contamination in spike-sorted electrophysiology data" and both reviewers agree that the study provides a valuable evaluation of the quality of spike sorting from extracellular electrophysiology recordings. Specifically, the reviewers agreed that the authors provide a better understanding of how ISI violations and spike cluster contamination relate to each other. Both reviewers indicated a number of changes should be made to improve the clarity of the manuscript, including providing more details of the equations and simulations employed in the manuscript. These issues, and others, are detailed below, which you should find useful in revising your manuscript, if you decide to resubmit it to eNeuro or to another journal.

1) The Poisson assumption.

A major component of the simulations that the authors use is that neurons are Poisson. Do the results in the present manuscript depend on that assumption?

2) Line 201: "The dependence of this phenomenon on...": the meaning of this sentence is unclear. Please explicit as early as possible in the text what you mean by FP-to-FP violation (as well as FP-TP, TP-TP being obviously ruled out). One possibility would be to write it near the example with the arrows in Panel C(right) of Fig. 1.

3) Even if "rate" is customarily used both in the sense of "per total number", e.g., falsely detected spikes/total spikes (FDR) and in the temporal sense of "per second", here, the usage of one and the same word in different meanings quite confusing and the reader should be warned/helped here. We suggest using two different words: if "rate" shall be used for temporal frequency (as in equation (2), then it shouldn't be used for ISI_v and FDR (even if, unfortunately, it appears in the customarily used acronym). Alternatively (and perhaps preferably), the term "frequency" or similar could be used when "rate" is used in the sense of "per second".

4) Inclusion of details in the Methods section

Line 227: The intermediary steps that are taken to link equations 2 and 3 need to be described, perhaps in the Methods section. It might be more logical to permute the order of equations 2 3 4 5 6 as 2 5 6 4. It might also help to explicitly state that the Rs in eq. 2 are to be understood as the parameters of the Poisson processes generating the spike trains (and not as the result of an actual measurement).

Line 114: Please describe the parameters N, Rt, n and RFP in the Methods.

The description of how the inhomogeneous Poisson spike-trains were constructed needs more detail. How were the PSTHs selected exactly?

Line 114-126: The process of simulating the cluster populations is unclear. Is the process such that first target FDR and Rt are sampled, then for a given randomly sampled N, the PSTHs are sampled and scaled to achieve the target FDR and Rt? This section would benefit from significant revision.

We would appreciate a more detailed explanation and maybe a little help for the derivation of eq. 13. This could perhaps be put into the Methods section or into an Appendix in order not to hamper the flow of the main text.

5) What exactly is presented in Figure 2C? The figure legend and the manuscript text state that it shows simulated ISI distributions. How might it be possible to simulate the spiking activity of a cluster with ∞ contaminating neurons. For simulations, to the best of our understanding, N cannot be unbounded.

6) The process for estimating RFP, a necessary parameter for applying the authors' method in real data, should be described in detail,

Details:

1) A table summarizing the definitions of the various symbols (with their units) would be of great help sparing the reader the hassle of searching the text.

2) Eq. 13: the index "i" disappears.... I suppose it runs over the elements of Rfp?

3) Was the ISIv in Fig. 4A was chosen to be 0.5 % because of customary usage? Maybe this could be specified?

4) Why, in the table, was the homogeneous equation used in some cases and in others not?

5) Line 360: "Median estimated FDRs were consistently lower than mean estimated FDRs." This is not true for Juavinett (last line of the table).

6) Figure issues:

Fig 2:

It would be preferable to use the same y-axis scale in all panels, except in the leftmost one in A. Even in A, however, it would help to overlay, in high transparency, the ISI counts for the refractory period of the leftmost panel onto the other two panels - rescaled to their same scale. In order to be able to directly compare the counts between the different conditions.

Please add the information that the model used was the inhomogeneous one to the legend of Fig. 2D.

Fig. 3:

Please specify (preferably in the legend), for each panel, whether firing was homogeneous or inhomogeneous, as well as what was the used equation.

Fig. 4:

What were the N and RFP values that were used for computing the analytical predictions in Figure 4? The figure legend would be a good place to show them.

7) Lines 312-319: I find this part a bit oddly written. I'd put the sentence "when inhomogeneous..." first. Only afterwards I'd mention the fact that, when TP and FP spike rates do not covary in time, the homogeneous equation does a good job, even if firing was inhomogeneous. BTW, do you have any idea why this is the case?

8) It seems to me that you didn't define RMSE. I would do so, even if this is quite a standard acronym.

9) Minor suggestions for language

Line 14: "spikes are misassigned to the wrong cluster": One might write "spikes are assigned to the wrong cluster"

Line 286: as in the rest of the manuscript, keep "data" plural

## Author Response

Response to Reviewers Summary - Review 2 We thank the Reviewers for their helpful additional feedback and have addressed their two comments below.

Major Issues 1. Mathematical notation. The mathematical notation should be uniform throughout the manuscript. The scalar product between two vectors is sometimes written with a dot and sometimes without a dot. Further, it appears at times that the dot is used to indicate multiplication between scalar quantities. In addition, it is unclear what the basis was for dropping the index in equation 13.

This is a good point, and one that we had not considered when adding multiplication dots for visual clarity. All dots in mathematical notation have now been removed in favor of implicit multiplication or "multiplication by juxtaposition" except when representing the dot product between two vectors, and parentheses have been added when needed to enhance visual clarity.

In regards to Eq. 13, we agree that the operations being performed in this equation may be a little unclear. It's our understanding that a simple summation of a 1D vector across all elements is commonly represented through omission of summation index and bounds. This summation divided by n just corresponds to calculating the average value of f ^_FP or f ®_FP (fFP-bar). It's expanded in Eq. 13 to make clear that this average is taken after first scaling f ^_FP by the magnitude |f ^_FP | obtained in Eq. 12. To make these points clearer, we have added a second equality in Eq. 13: f ®_FP/f ®_t .

2) Computing ISIv. In figure 2, the authors show simulations with different FDR% (columns), but similar ISIv, but the basis for the ISIv was not clear. In particular, the plots seem to be in contradiction with the numbers. ISIs falling within the red/pink rectangle correspond to ISI-violations. They are supposed to be 0.5% of all ISIs, for all spiking frequencies (f_t = 20Hz, 5Hz and 4Hz). However, the ratio between the mean taken over the red rectangle and that taken over the rest of the distribution does not look the same in the three panels. This ratio seems much larger at 4hz (near 1/3?) than at 20Hz (near 1/10?), whereas it should stay the same for all conditions. Also, in C (leftmost panel in particular), this ratio seems to be more like 5% and not 1%. Is there a problem with the computation of ISIv? Addressing this issue is important because a central claim of the manuscript is that that ISI violations can stay the same under different spiking rates, even if the FDR is radically different.

This is a very valid concern, and we thank the reviewers for drawing our attention to a potential source of confusion about a central conclusion of our work in Fig. 2A. The reason that ISI violations may appear to constitute a smaller proportion of total ISIs in this panel for higher firing frequency conditions is because these conditions have lower average ISIs and all conditions are plotted within the same 0-25 ms ISI domain. The average ISI for a 20 Hz cluster is 50 ms, while for a 4 Hz cluster, it's 250 ms. This means that, for a 20 Hz cluster, there are proportionally many more ISIs < 25 ms than for a 4 Hz cluster. Therefore, ISI violations may appear to correspond to a smaller proportion of total ISIs in the 20 Hz cluster's histogram, but calculated across the full possible range of ISIs, ISI violation rates are the same across conditions. FDRs and firing frequencies were specifically chosen and verified through simulation to ensure this would be the case. We have added two sentences clarifying this point in the caption for Fig. 2.

Summary - Review 1 We thank the Reviewers for their close reading of our manuscript, as well as their helpful comments which will both improve readability as well as clarify important methodological details. We are pleased that the Reviewers found that our study provides "a valuable evaluation of the quality of spike sorting from extracellular electrophysiology recordings," as well as "a better understanding of how ISI violations and spike cluster contamination relate to each other." We have addressed each comment made by the Reviewers below, in the order they were provided within the synthesis of reviews.

General Comments 1. The Poisson assumption.

A major component of the simulations that the authors use is that neurons are Poisson. Do the results in the present manuscript depend on that assumption? This is an excellent question and one that we had not fully considered while preparing the initial submission. After simulating non-Poisson point processes across a range of physiological coefficients of variation and then attempting to predict FDR from observed ISIv, we can confirm that our results do not depend on Poisson simulation of neural spiking. FDR predictions remained highly accurate (RMSE = 0.01-0.02) when attempting to predict FDR for neural spiking simulated with ISIs drawn from gamma, inverse Gaussian, and log-normal distributions with coefficients of variation of 0.5, 1, and 2. These results imply that our model likely generalizes to any renewal process that produces independent and identically distributed ISIs with a physiological coefficient of variation. These results have been organized and presented in Extended Data Figure 3-1. Discussion of the model's ability to generalize to non-Poisson point processes, referenced to Extended Data Figure 3-1, is now provided in the text at the start of Materials and Methods, the section of the Results discussing Fig. 3D, and in the Discussion.

2. Line 201: "The dependence of this phenomenon on...": the meaning of this sentence is unclear. Please explicit as early as possible in the text what you mean by FP-to-FP violation (as well as FP-TP, TP-TP being obviously ruled out). One possibility would be to write it near the example with the arrows in Panel C(right) of Fig. 1.

The TP-to-FP and FP-to-FP violations in Fig. 1C have now been labeled appropriately in the figure with a brief explanation also included in the figure legend. A figure citation for Fig. 1C has also been added to the sentence starting "The dependence of this phenomenon on..." after mentioning "FP-to-FP violations." 3. Even if "rate" is customarily used both in the sense of "per total number", e.g., falsely detected spikes/total spikes (FDR) and in the temporal sense of "per second", here, the usage of one and the same word in different meanings quite confusing and the reader should be warned/helped here. We suggest using two different words: if "rate" shall be used for temporal frequency (as in equation (2), then it shouldn't be used for ISI_v and FDR (even if, unfortunately, it appears in the customarily used acronym). Alternatively (and perhaps preferably), the term "frequency" or similar could be used when "rate" is used in the sense of "per second".

In all instances where rate is used in the sense of "per second," e.g. firing rate, true positive spike rate, this term has been replaced with frequency. The symbols in figures and equations have also accordingly been adjusted. Instances where rate is used in the sense of "per total number," have been preserved as rate, e.g. inter-spike-interval violation rate, false discovery rate.

4. Inclusion of details in the Methods section Line 227: The intermediary steps that are taken to link equations 2 and 3 need to be described, perhaps in the Methods section. It might be more logical to permute the order of equations 2 3 4 5 6 as 2 5 6 4. It might also help to explicitly state that the Rs in eq. 2 are to be understood as the parameters of the Poisson processes generating the spike trains (and not as the result of an actual measurement).

A section has been added in the Appendix outlining the derivation of Eq. 6 from previous equations in greater detail. The equations have also been permuted such that the substitutions and relationships needed to derive Eq. 6 are introduced before deriving Eq. 6. Finally, it has been clarified in the text that fTP and fFP represent unseen parameters of spike train generation that are not measured experimentally.

Line 114: Please describe the parameters N, Rt, n and RFP in the Methods.

These parameters are now described in Table 1, which defines and provides units for all the symbols used throughout the manuscript.

The description of how the inhomogeneous Poisson spike-trains were constructed needs more detail. How were the PSTHs selected exactly? PSTHs were selected randomly from among all clusters contained within a dataset. The trial-averaged PSTH was then taken to be the time-varying Poisson rate used to generate spike trains. Longer simulated recording times were attained through repeated simulation of these PSTHs. This is now explained in greater detail in the section titled "Monte Carlo simulation of cluster populations" in Materials and Methods.

Line 114-126: The process of simulating the cluster populations is unclear. Is the process such that first target FDR and Rt are sampled, then for a given randomly sampled N, the PSTHs are sampled and scaled to achieve the target FDR and Rt? This section would benefit from significant revision.

The process described by the reviewers is correct, although this workflow was difficult to understand in the previous manuscript version. As suggested, the section titled "Monte Carlo simulation of cluster populations" in Materials and Methods has been expanded and reworked to improve the clarity and flow of these points. Specifically, each step in the process is now presented with a numeric label in the order it was performed, and significant additional detail has been added at each stage.

We would appreciate a more detailed explanation and maybe a little help for the derivation of eq. 13. This could perhaps be put into the Methods section or into an Appendix in order not to hamper the flow of the main text.

Eq. 13 describes the relatively straightforward calculation of FDR once the magnitude of f ^_FP has been determined using Eq. 12. Given that Eq. 12 is much more complex and intermediate parts of its derivation were omitted in the original submission, we assume that the reviewers were referring to Eq. 12 in this comment. Accordingly, a section has been added in the Appendix outlining the derivation of Eq. 12 from previous equations in greater detail to guide interested readers through intermediate steps from Eq. 10 to Eq. 12.

5. What exactly is presented in Figure 2C? The figure legend and the manuscript text state that it shows simulated ISI distributions. How might it be possible to simulate the spiking activity of a cluster with ∞ contaminating neurons. For simulations, to the best of our understanding, N cannot be unbounded.

The reviewers are correct that a cluster with an infinite number of "contaminant" neurons cannot be directly simulated. This was a convenient shorthand we used to describe the limiting case where there are as many contaminant neurons as FP spikes. In that case, any FP can produce an ISI violation with any other FP. This limiting case can be simulated using a single contaminant neuron with no refractory period. We have now clarified this point at the start of Materials and Methods, in the figure legend for Fig. 2, as well as when the concept of infinite contaminant neurons is introduced in the Results (Analytical model of the relationship between ISIv and FDR).

6. The process for estimating RFP, a necessary parameter for applying the authors' method in real data, should be described in detail.

To facilitate a more detailed explanation of the process by which unknown model parameters are estimated as well as improve flow, the section in Materials and Methods previously titled Estimation of FDR in publicly available electrophysiology datasets has now been split into two sections titled Estimating unknown cluster parameters and Estimation of FDR in simulated cluster populations and electrophysiology datasets. The first contains a rigorous explanation of how all parameters needed to predict FDR can be estimated, including f ^_FP.

Details 1. A table summarizing the definitions of the various symbols (with their units) would be of great help sparing the reader the hassle of searching the text.

Table 1 has now been inserted at the start of Materials and Methods, which defines and provides units for all the symbols used throughout the manuscript.

2. Eq. 13: the index "i" disappears... I suppose it runs over the elements of RFP? The index "i" in this equation is meant to run over the elements of fFP as suggested. Since the only term in the summation symbol is the vector fFP, and therefore the only way to conduct this summation is over the elements of fFP, we have removed the index and summation bounds from the sigma symbol in this equation (Eq. 13).

3. Was the ISIv in Fig. 4A chosen to be 0.5% because of customary usage? Maybe this could be specified? The choice of 0.5% was somewhat arbitrary, although we note that 0.5% has sometimes been used as threshold for considering units well-isolated and is a commonly found % ISIv in spike-sorted electrophysiology data. This explanation is now included in the figure legend for Fig. 4A.

4. Why, in the table, was the homogeneous equation used in some cases and in others not? The homogenous equation was used for datasets acquired during behaviors that were not trial-based in structure. In this case, we could not compute trial-averaged firing rates, which we used for the generation of inhomogeneous Poisson spike trains. One could in principle estimate the time-varying (inhomogeneous) Poisson rate from single-trial data, but we found that the degree of temporal smoothing used to estimate firing rates had a large effect on predicted FDR. This is discussed in detail in Materials and Methods. A brief explanation of the usage of the homogeneous equation has been added in Line #, as well as a pointer to Materials and Methods.

5. Line 360: "Median estimated FDRs were consistently lower than mean estimated FDRs." This is not true for Juavinett (last line of the table).

The dataset from Juavinett et al. not having a lower median than mean estimated FDR has been clarified at line #.

6. Figure issues:

Fig. 2: It would be preferable to use the same y-axis scale in all panels, except in the leftmost one in A. Even in A, however, it would help to overlay, in high transparency, the ISI counts for the refractory period of the leftmost panel onto the other two panels - rescaled to their same scale. In order to be able to directly compare the counts between the different conditions. Please add the information that the model used was the inhomogeneous one to the legend of Fig. 2D.

In each section of Fig. 2 except for Fig. 2A, the y-axis maximum has been set to the same value and the histograms have been scaled accordingly. In Fig. 2A, the right two panels have been set to the same y-axis scale, and their outlines have been superimposed to scale on the leftmost panel in high transparency to facilitate direct comparisons of the three conditions. This superimposition is described in the figure legend as well. Fig. 2D doesn't exist; we assume this comment refers to Fig. 2B and have also added a sentence in the figure legend describing whether each section was simulated with homogeneous or inhomogeneous firing.

Fig. 3: Please specify (preferably in the legend), for each panel, whether firing was homogeneous or inhomogeneous, as well as what was the used equation.

A sentence has now been added at the end of the figure legend denoting for each panel whether simulated firing was homogeneous or inhomogeneous and which equation was used for FDR predictions.

Fig. 4: What were the N and RFP values that were used for computing the analytical predictions in Figure 4? The figure legend would be a good place to show them.

Possible values for ft, N, and f ^_FP are now listed in the figure legend for Fig. 4B and Fig. 3D along with a pointer to Materials and Methods where the process of simulating cluster populations is described in greater detail.

7. Lines 312-319: I find this part a bit oddly written. I'd put the sentence "when inhomogeneous..." first. Only afterwards I'd mention the fact that, when TP and FP spike rates do not covary in time, the homogeneous equation does a good job, even if firing was inhomogeneous. BTW, do you have any idea why this is the case? These sentences have been reordered as suggested. Additionally, explanation has been added as to why the homogeneous equation returns good predictions for TP and FP spike rates that do not covary in time, even if their firing is inhomogeneous. Briefly, when cov(fTP, fFP) = 0, their concentration in time is, on average, equivalent to the homogenous case, meaning the odds of an ISI violation occurring are unchanged. In this special case, the homogeneous and inhomogeneous predictions agree.

8. It seems to me that you didn't define RMSE. I would do so, even if this is quite a standard acronym.

Root mean square error (RMSE) is now defined both times it appears in figure legends (Fig. 3D, Fig. 4B) and the first time it appears in the text (Line #).

9. Minor suggestions for language Line 14: "spikes are misassigned to the wrong cluster": One might write "spikes are assigned to the wrong cluster" In Line #, "misassigned" has been replaced with "assigned." Line 286: as in the rest of the manuscript, keep "data" plural "Data" has been consistently pluralized throughout the manuscript.
