# Supplementary figures and images for: Assessing Cross-Contamination in Spike-Sorted Electrophysiology Data
Source: eNeuro. 2024 Aug 20;11(8):ENEURO.0554-23.2024. doi: 10.1523/ENEURO.0554-23.2024 (PMC11368414; doi:10.1523/ENEURO.0554-23.2024)

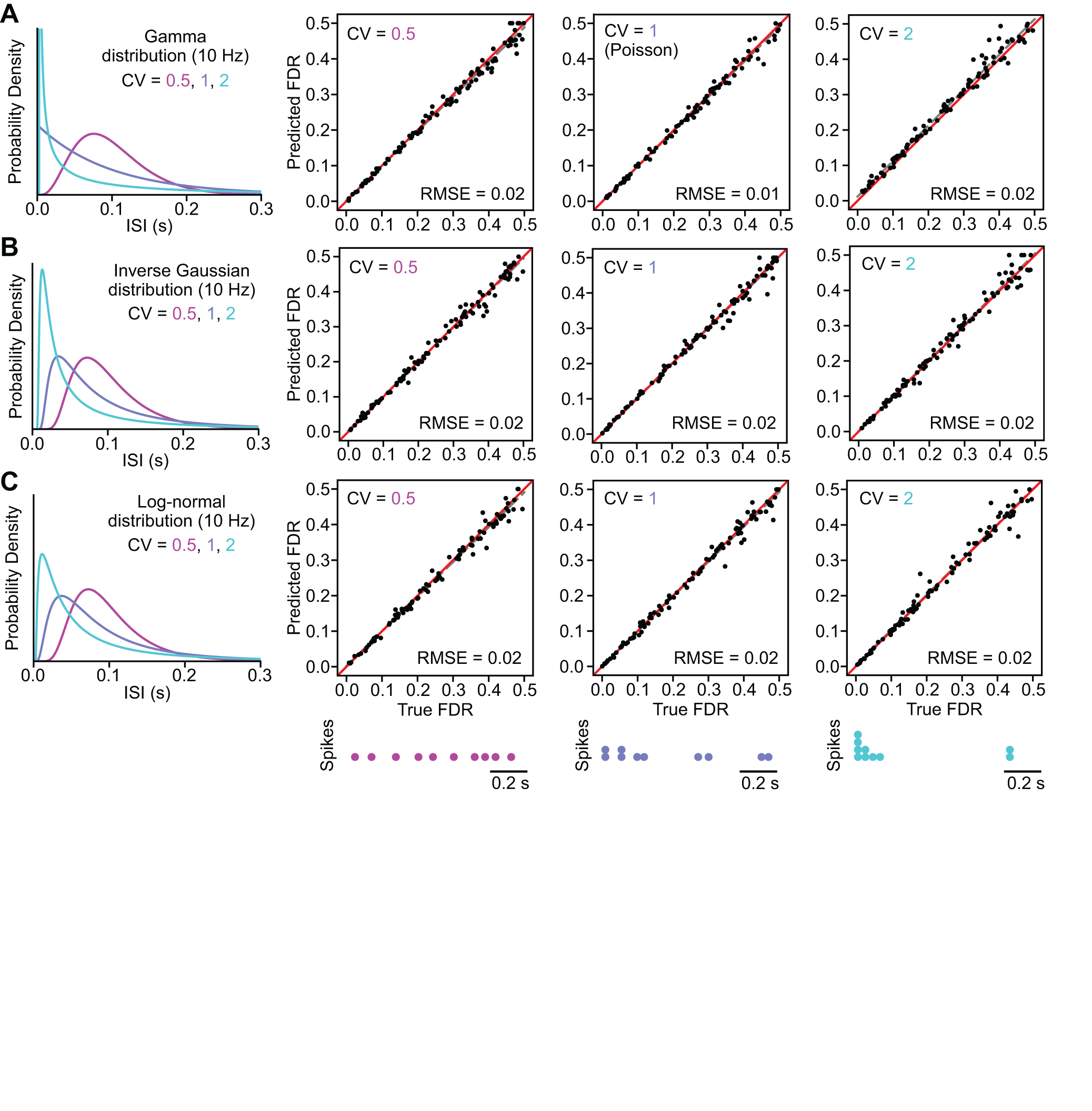

Supplement: Figure 3-1 — Prediction of FDR in non-Poisson point processes. Prediction of FDR from observed ISIv when simulating neural spiking by drawing ISIs from gamma distributions (A), inverse Gaussian distributions (B), or log-normal distributions (C). 100 total clusters simulated in each panel across a range of physiologically relevant underlying neuronal characteristics. Total firing frequency was varied between 4 and 20 Hz, N was varied between 1 and 10, and f^FP was obtained by averaging across other clusters (see Materials and Methods for more details). Coefficient of variation (CV) of ISI distributions varied from 0.5 to 2. Red line is the unity line, or perfect concurrence between predicted and true FDR; dashed gray line is the line of best fit. Root mean square error (RMSE) calculated with respect to the unity line. Simulated firing was homogeneous and the model used for FDR predictions was also homogeneous (Eq. 9). A gamma distribution with CV = 1 is identical to an exponential distribution, producing a Poisson point process. Bottom raster plots show gamma distributed spiking of an example 10 Hz neuron at various CVs. Stacked points indicate spikes occurring in quick succession. Download Figure 3-1, TIF file. [file eneuro-11-ENEURO.0554-23.2024-s001.tif]
